# Supporting the consumption and co-authoring of locative media experiences for a rural village community: design and field trial evaluation of the SHARC2.0 framework

**DOI:** 10.1007/s11042-016-3515-y

**Published:** 2016-04-14

**Authors:** Keith Cheverst, Helen Turner, Trien Do, Dan Fitton

**Affiliations:** 1 Lancaster University, Lancaster, UK; 2 University of Central Lancashire, Preston, UK

**Keywords:** Locative media, Field trial, Rural community, Local history

## Abstract

Locative Media Experiences (LMEs) have significant potential in enabling visitors to engage with the places that they visit through an appreciation of local history. For example, a visitor to Berlin that is exploring remnants of the Berlin Wall may be encouraged to appreciate (or in part experience) the falling of the Berlin wall by consuming multimedia directly related to her current location such as listening to audio recordings of the assembled crowds on 10th November 1989. However, despite the growing popularity of enabling technologies (such as GPS-equipped smart phones and tablets), the availability of tools that support the authoring of LMEs is limited. In addition, mobile apps that support the consumption of LMEs typically adopt an approach that precludes users from being able to respond with their own multimedia contributions. In this article we describe the design and evaluation of the SHARC2.0 framework that has been developed as part of our long-term and participatory engagement with the rural village of Wray in the north of England. Wray has very limited cellular data coverage which has placed a requirement on the framework and associated tools to operate without reliance on network connectivity. A field study is presented which featured a LME relating to Wray’s local history and which contained multimedia content contributed by members of the community including historic photos (taken from an existing ‘Digital Noticeboard’ system), audio-clips (from a local historian and village residents) and video (contributed during a design workshop). The novelty of our approach relates to the ability of multiple authors to contribute to a LME in-situ, and the utilisation of personal cloud storage for storing the contents associated with a multi-authored LME.

## Introduction

The term Locative Media was introduced by Karlis Kalnins in 2003 [18] and since then the growing popularity of GPS equipped smartphones and tablets has served to increase the opportunity for Locative Media Experiences (hereafter abbreviated to LMEs). While various definitions exist, the following definition provided by [3] is apt for this article:

“…the locative media that is of most immediate concern is that made by those who create experiences that take into account the geographic locale of interest, typically by elevating that geographic locale beyond its instrumentalized status as a ‘latitude longitude coordinated point on earth’ to the level of existential, inhabited, experienced and lived place. These locative media experiences may delve “into” the historical surface of a space to reveal past events or stories (whether fictional, confessional or standing on consensus as factual)…”

LMEs have significant potential in enabling visitors to engage with the places that they visit through an appreciation of local history. For example, a visitor to Berlin that is exploring remnants of the Berlin wall may be encouraged to appreciate (or in part experience) the falling of the Berlin wall by consuming multimedia directly related to her current location, e.g. listening to audio recordings of the assembled crowds on 10th November 1989 or viewing images that clearly show how the crumbled remnant in front of them appeared against the same backdrop of buildings before it was demolished.

It is important to note that LMEs need not be restricted to having a single author. Indeed, one of the seminal Locative Media projects, the Urban Tapestries project [34], developed a prototype to explore the utility of an open approach to the contribution of content that:

“affords its users a novel way of authoring their own experience of inhabiting the cityspace and communicating it to others via an album of memories structured around sound” and continue to state that: “Urban Tapestries relies on the co-creation of its own content by its users, rather than the consumption of pre-prepared content”.

**Fig. 1:**
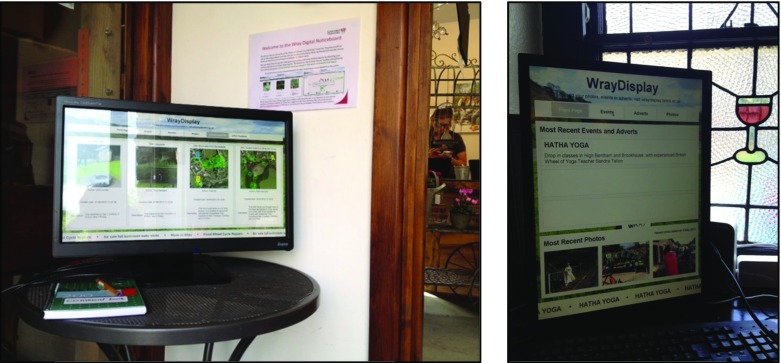
Deployments of the *WrayDisplay* at Wray’s Garden Centre and the Village pub

The definition by [3] and the open approach taken by Urban Tapestries resonates strongly with the motivations of the SHARC (*Shared Curation of Local History in a Rural Community*) research project in which we seek to develop tools that enable residents of a rural village named Wray (situated in the north of England) to co-curate LMEs relating to the village’s local history. The project builds on our on-going longitudinal ‘research in the wild’ [10] with the village and in particular our research involving the design and long-term deployment of a situated digital display system. This system, known as the *WrayDisplay*, supports the sharing of photos submitted by village residents [51]. Since 2006, the touch-screen displays have been deployed in key places within the village such as the village Post Office, local café, village hall and, most recently, the village pub (see Fig. 1, right) and Garden Centre (see Fig. 1, left). The current version of the system enables residents and visitors to view LMEs associated with the village and to download these onto their smartphone devices.

**Fig. 2:**
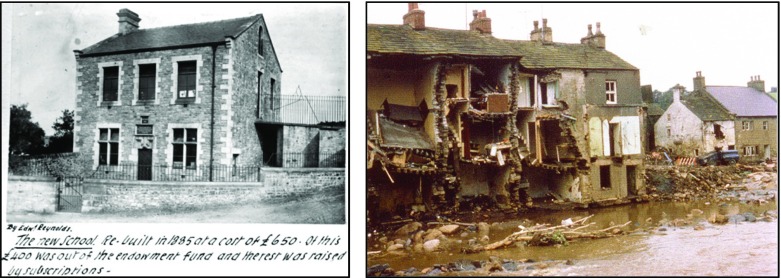
Historic photos of POIs in Wray submitted by residents to the *WrayDisplay* system: Wray School (*left*) and damage caused by the Wray Flood of 1967 (*right*)

Our motivation for supporting LMEs relating to, in particular, Wray’s local history reflects the interest shown by the community in the photo content submitted to the *WrayDisplay* system. Indeed, a recent analysis of the photo content revealed that a significant portion of the 2800 images relate to the Cultural Heritage and Local history of Wray [13]. Furthermore, Wray contains a number of key Points of Interest (POI) that have significant relevance to Wray’s local history and photos of these POIs featured strongly, especially showing their appearance from the past. For example, one key POI is Wray’s 17th Century School house (see Fig. 2, left) which is the setting of numerous stories and tales including failed attempts to purchase and close the school. Also relevant to Wray’s history are a number of key Events of Interest (hereafter referred to as EOIs) such as the flood of 1967 which caused widespread damage to properties in the village (see Fig. 2, right).

In this article we describe the design and field-trial evaluation of the tools and mobile apps developed through the SHARC project in order to support the co-authoring and consumption of LMEs. The following scenario illustrates one way in which a visitor could interact with the *WrayDisplay* system in Wray and consume LMEs using the Android SMEP app (*SHARC Mobile Experience Player*) and, while consuming the LME, add her own multimedia responses, which can then become part of the LME:

A visitor, Jane, arrives at Wray’s Garden Centre and interacts (using touch) with photos that have been uploaded to the *WrayDisplay*. On viewing a series of old photos of Wray School she decides to download onto her smartphone a LME created by a local historian that includes Wray School as part of a short circular walk around the village. The village has no cellular coverage but the Garden Centre has free Wi-Fi and so she uses this to download the smartphone app (SMEP) and the LME (including all associated media files). The SMEP app displays a map highlighting the route, her current location (sensed by the phone’s GPS) and several POIs including Wray School. Jane decides to head directly to Wray School and as the building comes into view her smartphone vibrates and Jane looks down to view a scrollable media pane containing several old photos of the school, and two pieces of audio. The first audio clip is from the local historian and provides a brief description regarding the significance of the school in Wray’s history. However, the second piece of audio is actually a narrative recorded by Henry, a resident of the village, in which he describes the role of his great grandfather in thwarting attempts to close and sell off the school building in 1898. Jane is impressed by Henry’s story and decides to respond with her own photo and short piece of audio. On returning to the Garden Centre Jane uploads her response to the LME via her personal Google Drive storage on the understanding that her response will be incorporated into the LME if the historian approves it.

The above scenario illustrates the co-authoring aspect that features strongly in our approach. In more detail, while the LME was initially authored by the local historian, multimedia content had subsequently been added by a local resident and then by Jane herself. The scenario also highlights a key constraint imposed by the intended rural deployment environment, i.e. the lack of cellular data and reliance on limited Wi-Fi hotspots. This connectivity issue has imposed a significant requirement on the developed tools and apps. For example, the SMEP app has been designed to download all locative media files to a user’s device before they commence the LME.

The scenario also illustrates the use of personal cloud storage for storing the media associated with LMEs. For example, the designer/creator of the LME (i.e. the local historian, Sarah) would store the photos and her audio file on her cloud storage. Similarly, the villager in the scenario, Henry, would store his audio file and contributed photo on his own cloud storage.

The SMEP app is one component of the SHARC2.0 framework which also includes tools (both web-based and mobile) to support the authoring of LMEs. The SHARC2.0 framework significantly extends and revises an earlier version of the SHARC framework (presented in [12]) by supporting multiple personal cloud storage solutions and additional/enhanced tools that support the mobile authoring and consumption of LMEs. A further component of the framework is responsible for supporting the browsing of authored LMEs and this component is incorporated into the current *WrayDisplay* software. The novelty and contribution of our approach relates to the supported functionality that enables multiple authors to contribute multimedia to a LME in-situ, and the utilisation of personal cloud storage for storing the multimedia contents associated with a co-authored LME. Furthermore, the results of the field trial conducted in the village of Wray provide insights into the issues associated with our approach such as sustainability and participation.

The field trial evaluation is central to our adoption of a research in the wild approach in which “*researchers are experimenting with new technological possibilities that can change and even disrupt behaviour. Prototyping in the wild is on the rise where objects*, *artefacts*, *and other inventions are assembled and then tried out in the settings for which they are envisioned*” [48]. This in-situ approach to evaluation contrasts strongly with Lab-based evaluation [30, 31, 49], which, while providing control over factors such as the weather, are poor at capturing context of use [49] and providing ecological validity [7]. Indeed, in reflecting upon the advantages of carrying out in-situ studies Kjeldskov *et. al*. describe how such studies support “*the gathering of large amounts of rich and grounded data*, *and a high level of ecological validity*” [31]. The main disadvantage of in-situ studies is that they are typically more expensive to resource [30] especially where such studies involve the long-term deployment of interactive systems ‘in the wild’ [37].

The remainder of this paper is organized as follows: Section 2 describes the background and related work in terms of LMEs and tools that support their authoring. Section 3 presents an overview of the SHARC2.0 framework. Following this, Section 4 describes a field trial that took place in Wray (involving both visitors and residents) and featuring a LME based on a route contributed by a local historian that includes media contributed by village residents. Next, Section 5 presents additional detail on the way in which multimedia content associated with an LME is propagated via the SHARC2.0 framework. Section 6 provides a discussion. Finally, Section 7 draws conclusions from the research.

## Related work

Our coverage of related work focuses on three areas: i) documented LMEs and associated projects within the research literature that relate to communicating a place’s local history or heritage, ii) tools and frameworks that support the authoring of LMEs and the documented requirements for such tools, iii) projects that while not framed as incorporating LMEs are location-based and incorporate content relating to a place’s local history in order to facilitate community engagement.

### LMEs relating to local history or heritage

One of the earliest examples of a LME related project (dating from 2002 and actually pre-dating the locative media term) was the ‘34 North 118 West’ project (http://34n118w.net/). The project was conceived as an experimental art work and coupled location sensing (GPS in this case) with mobile computing devices in order to provide users with a ‘locative narrative’ in which the mobile device would push audio narratives relating to the history of places they passed by in Los Angeles. It is important to note that ‘34 North 118 West’ project is an early example of an LME in which contribution of content by users other that the artist or designer is deliberately not supported, i.e. the artist or designer is solely responsible for scripting the content incorporated into the LME.

A contrasting approach is illustrated within the Urban Tapestries project [34], which has a public or open approach to the contribution of content. Indeed, [34] describes a motivating scenario in which:

“… as a user moves through the streets passively accessing the location based information, this inspires them to add their own locations, favourite threads and leave messages for others”.

The SHARC project described in this article fosters a similarly open approach whereby the community in which the system is deployed can contribute to an LME that has been authored.

Perhaps the most seminal LME documented in the research literature is the Riot! 1831 experience by [42] which is based on an interactive play of the actual riots that took place across England in 1831. In common with the experiences associated with the’34 North 118 West’ project, the Riot! 1831 experience relied solely on audio media, where a user (or consumer) would walk around Queen Square, Bristol, with a set of headphones, listening to the sound files triggered by changes in the user’s sensed location. The use of engaging audio in the Riot! 1831 LME enabled users to become absorbed in the experience, e.g. “*you could hear the rioters*’ *voices as they plundered the surrounding buildings*, *the flames as buildings burn*…” [42].

This notion of allowing people to ‘lose’ themselves in the experience and the distraction it provides from everyday life is described as immersion by [26]. Referring specifically to computer games, [6] states that immersion is viewed as critical to game enjoyment; with [26] suggesting further that immersion is used to describe the degree of involvement with the game:

“Sometimes people find the game so engaging that they do not notice things around them … at such moments, all of their attention is focused on the game, even to the extent that some people describe themselves as being ‘in the game’.”

Following a user-trial of the Riot! 1831 LME, [42] describes the sense of immersion experienced by a father and son who took part in this experience. Similarly to the findings by [26, 42] identified a key result that immersion is a positive determinant for enjoyment (and vice versa), and further that:

“history coming alive is a special form of immersion that is related to the nature of the play and the historical setting … participants reported empathy with the people involved in the riots and a sense of walking in their footsteps.”

Furthermore, [42] observed that immersion is a transient state that can be fleeting or can last for several minutes and thus draw the conclusion that the circumstances that move people between immersive and non-immersive stages are an important consideration in designs of similar systems.

The design implications reported by [43] suggest that authenticity supports a sense of immersion by highlighting the significance of authenticity and setting for creating "magic moments" in an experience, particularly that: “*for history based Mediascapes*, *exposing authenticity is a powerful emotive mechanism to make people feel a connection to the past*”.

The findings from [43] imply that the fact that the riots took place in that actual location was an important factor in the enjoyment of the experience, and authenticity provides a sense of “coming alive” which allows people to view a place in a new way, supporting the findings for immersion as discussed by [42]. Further, results from the user trial in [42] suggest that the combination of different sound files and a constant background sound of a riot meant that the user was continuously ‘in’ the riot: “*There*’*s one thing to read about something and see pictures but when you actually hear the pain and the anguish* … *it makes you think well this actually happened*.”

The Riots of 1831 were also the centre of another study. The authors in [14] describe their study of two audio guides that utilised location-based triggering of audio to visitors wishing to learn about historic events (specifically, the 1831 Reform Riot) related to the city of Nottingham. In commenting on the LME featured in the study, participants described appreciating being able to relate the spoken narratives to real locations in the city and that it: “*enabled them to use their imagination to reconstruct the events of the time*.” [14].

A controlled study of reported immersion is also presented in [29] and involved tourists consuming a LME featuring a visual-narrative while visiting the city of Funchal, Madeira. The study found evidence that participants in the “*original narrative location*” reported a higher level of immersion, as well as heightened mental imagery (when compared to the “*controlled narrative location*”).

A review and analysis of the relationship between contemporary mobile apps (such as Yelp, Foursquare, etc.) and locative media is presented in [16].

### Tool and framework support for the authoring of LME’s

The research literature contains relatively few documented tools to support the authoring of LMEs. The Urban Tapestries project (introduced in the previous sub-section) introduced one of the first documented systems to support some form of mobile authoring tool [34]: “…*by combining mobile and internet technologies with geographic information systems*, *people could* ‘*author*’ *the environment around them*.”

The tool required connectivity via the mobile device’s cellular data connection and enabled non-technical users to attach text, audio clips or photos to specific addresses within central London, U.K.

A seminal authoring tool for locative-media is the Mediascape Framework, [24] also referred to as ’The Mobile Bristol Authoring Framework’ [25]. The framework comprised of tools to support the authoring, testing and playback of LMEs on windows Pocket PC mobile devices such as the Compaq iPAQ. It was designed to enable a wide-range of non-specialists (i.e. not necessarily programmers) to author sophisticated LMEs supporting a multitude of media formats, including; image, audio, video, Abode Flash movies and web pages. Furthermore, the LMEs produced could respond to a variety of contextual triggers, including location beacons utilising technologies such as Bluetooth. Later versions of the framework also supported off-line use by enabling content associated with LMEs to be stored on the memory card of a Pocket PC mobile device. Mediascape was used to author several LMEs in education, games, guided tours and historical reconstructions [25], including: Riot! 1831 [43], Tower of London [25] and Scape the Hood [44].

ColourMaps [15] enabled designers to author a location-based game by directly colouring over maps. It served the user with text, image and sound content dependent on their location on the map.

The LOTM Tool [23] supported the authoring of interactive mobile-learning trails, utilising technology such as GPS, Bluetooth and Image Recognition, to generate location-based trails of a variety of themes; including heritage trails to promote authentic and hands-on learning of the rich culture, historical and natural heritage of Singapore. The tool enabled users to add text, record videos, capture photos and make digital drawings while on the trails.

The M-Studio tool by [41] also supported the authoring of multimedia LMEs, specifically the creation of context-aware mobile stories. M-Studio provides the designer with a graphical manipulation interface for linking text, video, audio, or image content with a specific location.

In addition to the aforementioned tools that actually produce an LME as part of a rapid prototyping cycle, other tools have been developed that support designers in the early prototyping stages of developing an LME. Topiary [36] is a seminal tool that belongs to this category and enables designers to model the location of people, places and things, and demonstrate scenarios depicting location contexts. It has three workspaces; Active Map, Storyboard and Test where designers can model locations, create interface mock-ups and test their designs, respectively.

#### Requirements for supporting the authoring of LMEs

The literature reveals a number of requirements for creating and authoring location-based experiences.

The need to support the mobile, in-situ, authoring of locative media experiences is described in [53]. This research followed a co-design approach in enhancing the visitor experience to an historic country house, Chawton House, located in the South of England. This project had young children (in this case aged 10–11) as its target user group and also involved teachers and curators of Chawton House library in the co-design process. While the project did not result in the development of any tools, it produced a number of key findings from the co-design workshops carried out. One such finding, highly relevant to this article, was that: “*curators felt unable to tell stories naturally while not* in-situ” [53]. This signifies the importance of providing mobile authoring tools as the location often provides a trigger for storytelling. A phenomenon that is also emphasized in the method known as ‘fieldwork on foot’ [35].

Further to this, CAERUS [38], a context-aware mobile guide for tourist sites and educational centre’s, reports the value of using i) a handheld mobile tool for navigating the map, viewing content and/or capturing observations, and ii) an additional desktop application for the administration of location-based content. The authors of TOTEM in [28] support this view stating that it is “*naturally necessary for the content creators to leave their desktop workspace and go out into the real world*”.

### Location-based systems relating to local history and community engagement

In [22] the authors describe a mobile app called Lost State College (LSC) which is a mobile guide that provides official (i.e. commissioned by the app developers) historical photos and text-descriptions of a traditional college town in the Northeastern U.S. While using the app, users can also create and access user-generated content such as comments, uploaded photos, visits and likes. The associated user study aimed to explore how the social features, supported by the app, would reveal insights into participation around historic places. The study revealed that meaningful historic places evoked special attention from the participants, and that long-term residents tended to contribute more to the community heritage effort.

History Lines [32] is another example of a project facilitating community engagement but with a designed focus on location-based digital storytelling relating to community history. In this project, citizens from an urban master-planned development in Brisbane were provided with digital tools to capture and geo-tag community images with the purpose of producing a “*collective community memory*”.

One project that enabled users to explore shared ‘collective’ memories on public displays and via mobile apps was CLIO [46, 47]. The urban setting of Oulu, Finland, in which CLIO was deployed, contrasts to the rural village of Wray. CLIO enabled members of the general public to capture and share memories via text, images, audio, and videos. The submitted media could then be viewed on public displays (situated in both indoor and outdoor locations around Oulu) via a map-based interface or via a user’s personal android tablet or phone using the Layar Augmented Reality browser. While the approach of utilising both public displays and mobile devices has strong similarities with the approach adopted by SHARC, there are several notable differences between the two projects. In particular, the approach adopted by CLIO is one of tagging the media items submitted by users with context, such as location. In [46, 47] the authors do not refer to any adopted moderation strategy. Furthermore, the CLIO approach does not have the notion of LME ‘owners’ that can make decisions regarding the design and curation of the media content associated with their LME or indeed the route associated with their LME. Other notable differences include the fact that personal cloud storage is not used for storing media files and 3G/Wi-Fi connectivity is required for mobile access to the media repository.

Working with a rural Indian village, StoryBank [17] supported the creation and sharing of audiovisual stories. Users could create stories in a simple manner using their camera phone and these stories could then be transferred to a digital library attached to a village display. This approach removed any requirement for internet connectivity which was an important factor given the rural location.

Another notable project involving a rural community deployment was CrowdMemo [1] which was designed to support the preservation of local history and Cultural Heritage in the town of Arequito in Argentina. Using digital cameras, the community created videos about personal memories and associated them with locations in the town.

A community deployment based around the PlaceBooks system and involving a rural Welsh market town is presented in [21]. In common with our approach, the system was developed to support ‘off-line’ operation, given the poor connectivity available in the rural deployment setting.

## The SHARC2.0 framework

In this section we provide an overview of the SHARC2.0 software framework. The framework comprises four components that support the authoring, consumption and browsing of LMEs. Authoring is supported through the SLAT (SHARC Locative Media Authoring Tool) and SMAT (SHARC Mobile Authoring Tool) components. The consumption of LMEs is supported by the SMEP (SHARC Mobile Experience Player) component. Finally, the browsing of published LMEs is supported by the SPET (SHARC Public Exploration Tool) component.

One key feature of the framework is its use of personal cloud storage (currently support for both Dropbox and Google Drive has been implemented) for storing the multimedia content associated with LMEs. Our use of personal cloud storage was chosen to remove reliance on university storage [12].

Another significant aspect of the framework is that the associated mobile apps (SMEP and SMAT) are able to support operation in environments where internet connectivity is not necessarily available during their use ‘in the field’.

**Fig. 3:**
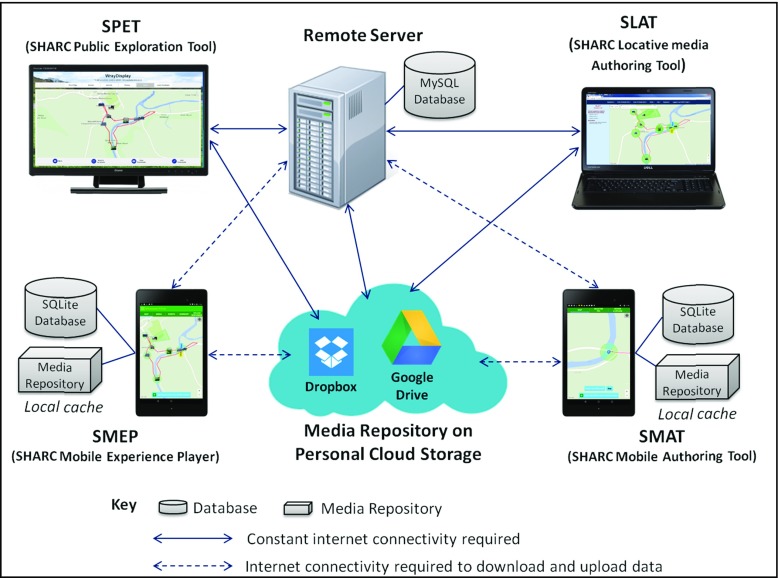
The interaction of components and cloud storage within the SHARC2.0 Framework

The relationship between each of the components and personal cloud storage is illustrated in Fig. 3. It can be seen from this figure how both SMEP and SMAT utilize local cache in order to store and access the multimedia content associated with a given LME during off-line use.

The following sub-sections describe each of the components in turn.

### SMEP: SHARC mobile experience player

The consumption of LMEs is supported by an Android app called SMEP which runs on Android smartphones and tablets running Android version 4.1 or newer.

SMEP utilises a push-based approach [8] to deliver multimedia content to the user based on their current location. In more detail, when a user enters the defined trigger zone associated with a POI then a notification sound and/or vibration is played and the locative media content is presented on a scrollable media pane. A user can then respond to the locative media content presented by submitting his/her own textual comment, audio/video clip or photo.

As introduced in section 1, the intended deployment domain of Wray village has (in common with many rural locations in the U.K.) no cellular data coverage. However, free Wi-Fi is available at a number of placed in the village including the two locations where *WrayDisplays* are currently deployed. In order to operate in this environment SMEP has been designed to download all locative media files before the user starts consuming the LME.

The app employs tabbed views in order to enable simple switching between a map view (where the user can view their You-are-here marker and see forthcoming POIs on a Google maps pane) and scrollable content related views. The map view tab is simply labelled as MAP. The scrollable content views consist of a POI media view (labelled MEDIA) and an EOI media view (labelled EVENTS). Other tab views allow users to view summary info (labelled SUMMARY) for the current LME (e.g. distance of route, number of POIs, etc.) and to review and upload their responses (labelled UPLOAD RESPONSES).

**Fig. 4:**
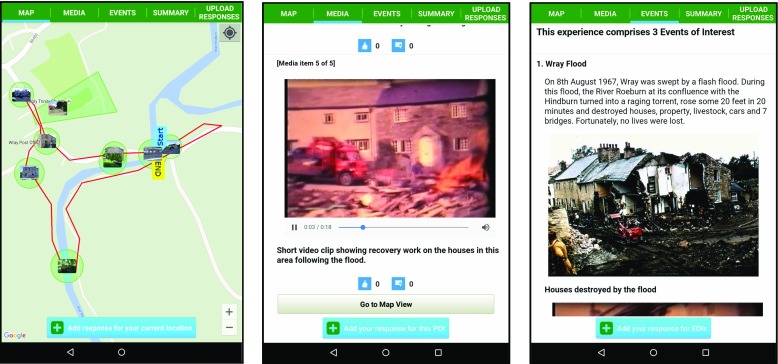
Illustrative screenshots of SMEP showing locative media content associated with Wray

Figure 4 illustrates sample screenshots from SMEP using content associated with Wray (further examples are provided in section 4).

The SMEP mobile app has undergone four main cycles of revision following testing at Wray village (the project’s intended place of deployment). In more detail, the SMEP player received four expert evaluations between July 2014 and February 2015 by experts in usability and interaction design. Rather than carrying out a lab-based usability study we acknowledge the greater value provided by undertaking the usability evaluation in-situ [49] and utilising the feedback provided as part of an iterative design approach to improve usability [33]. Within the MobileHCI literature there has been much discussion on the merits of carrying out studies in the field vs. in the lab [30, 31, 49] but given the importance of the setting to the LME, and its consumption, the decision to hold the expert evaluation sessions in in-situ, i.e. in Wray, had strong justification.

The four expert evaluations involved the same LME (called *Sarah*’*s Walk* and which is described section 4.1) as that used in the Field Trial (described in section 4). The graphical representation of the route is shown in Fig. 4 (left), Figs. 8 and 9. For each expert evaluation, the expert was provided with a Nexus 7 tablet already running the SMEP mobile app (with the locative media content already loaded) and then asked to Think-aloud [39, 40] while consuming the experience. Experts were accompanied by two of the authors. The expert evaluation sessions lasted between 65 and 80 min and the audio was captured and later transcribed.

The four expert evaluations formed a key part of the iterative prototype development process and proved invaluable in enabling the mobile app to attain the high level of usability and reliability appropriate for use in the field trial presented in section 4. To provide an illustrative example of this, the first expert evaluation involved an early version of the SMEP player prototype that did not feature separate tab views for displaying media content and showing the map view. During the expert trial, on approaching a trigger zone, the mobile app pushed content relating to the POI but the expert commented on her confusion regarding how to return to the map view. This feedback led directly to the introduction of the tab views. During the last expert evaluation, the expert commented that she would like access to summary information (e.g. the walking distance for the route and the number of associated POIs) regarding the LME being consumed and this led directly to the addition of a SUMMARY titled tab that presents this information.

It is also important to note that the SMEP app logs user interaction in order to enable the later triangulation of data produced during an evaluation study, e.g. triangulating qualitative data such as the user comments produced at a particular time/location with log data revealing the media items being pushed at the same time/location.

### SLAT: SHARC locative media authoring tool

The SLAT authoring tool is written as a web-based app to provide platform independent support for creating and editing LMEs. One key design decision underpinning the tool has been to make aspects of the authoring process relatively straightforward and to make the barriers to developing a LME as low as possible. One significant example of this relates to the creation of POIs within a LME. In more detail, a user can automatically define the geographic location of a POI by importing a geo-referenced image, e.g. a photo of Wray School that has been taken by a smartphone camera with the geo-tagging of photos function enabled. A default circular trigger zone (of 20 m radius) is then automatically associated with the POI which can be adjusted by dragging and dropping. Furthermore, the shape of the trigger zone can also be changed if required, e.g. to a polygon. The user can then chose to add additional multimedia content to the POI and subsequently change the ordering of the content. A simple emulator function is available in order to allow the author of an LME to virtually move the location of a You-are-here marker on the map canvas. When the marker enters the trigger zone associated with a given POI, this results in the playback of media associated with the given POI in a separate emulator window.

The SLAT tool can also be used to create an EOI, e.g. *Wray Flood* EOI, and to associate multimedia content with the EOIs and associate the EOI with POIs if appropriate, e.g. *Wray Flood* EOI could be associated with the *Flood Garden* POI. Furthermore, the tool enables the creation and editing of routes which can also be imported via KML (Keyhole Markup Language) files.

**Fig. 5:**
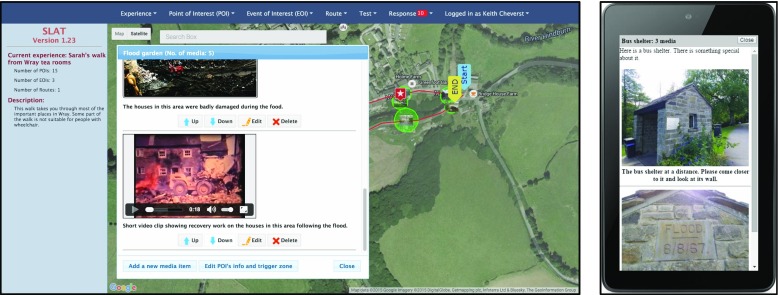
Illustrative screenshot of SLAT showing dialog for editing and reordering the media items associated with the ‘Flood Garden’ POI (*left*) and the emulator window (*right*)

An illustrative screenshot of SLAT is shown in Fig. 5. This figure highlights how the main window area is taken from a Google Maps pane that enables the author to view location of POIs and routes. The author can activate modal dialog windows to carry out specific functionality by either menu selection or by clicking an actionable object on the map canvas. For example, Fig. 5 shows the modal dialog window for the *Flood Garden* POI that provides the user with control over the media items associated with the POI. The user could have accessed this window by either clicking directly on the POI’s thumbnail on the map or by navigating through the “Point of Interest (POI)” menu item.

The SLAT authoring tool has received five expert evaluation sessions by experts in usability/interaction design between July 2014 and July 2015 resulting in modifications to the UI. The expert evaluation sessions took place in the university office of one of the authors and in four cases followed directly from the expert evaluation of the SMEP app in Wray. In the other case, the expert evaluation followed expert evaluation of the SMAT app on the university campus (see following sub-section). The feedback from the expert evaluations was ostensibly suggestions for improving usability, e.g. providing finer grained location controls when using the emulator, which were duly implemented.

**Fig. 6:**
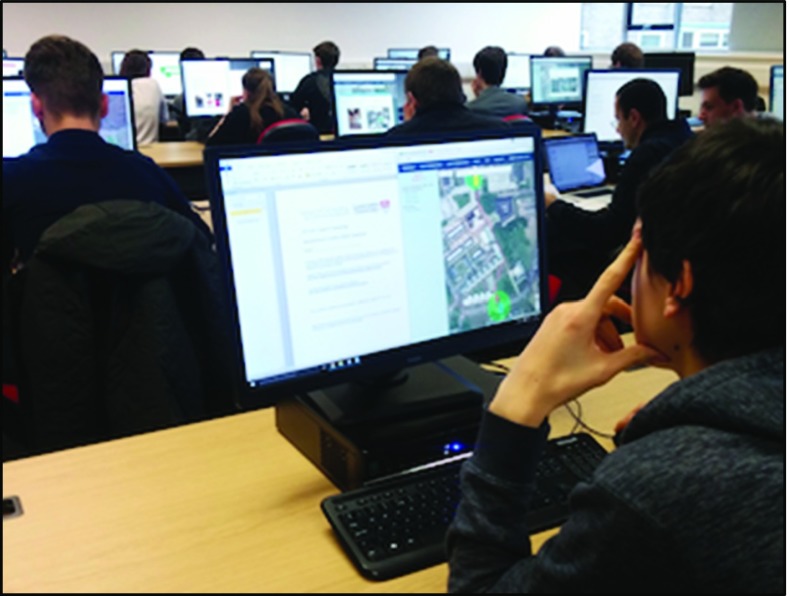
An undergraduate practical class involving the use of SLAT and SUS based evaluation

In addition, a usability study of SLAT was carried out involving 51 undergraduate students (enrolled on an ‘ICT for Creative Industries’ course) in November 2015 (see Fig. 6). The study was carried out as part of a weekly timetabled lab practical class (supervised by three of the authors) and involved a non-assessed exercise that required students to use SLAT in order to create a LME based around the university campus. The task involved creating two new POIs based on geo-referenced images and importing a KML file that represented a route between the two POIs. Students were also invited to create additional POIs and associated media items if they wished and were asked to complete an SUS (System Usability Scale) questionnaire [4] once they had completed the task. The SUS questionnaire utilises a simple 10-item scale giving a global view of subjective assessments of usability which yields a value between 0 and 100 [4]. It should be noted that practice with SUS was one of the intended learning outcomes of the session and students were required to calculate their SUS score using the scoring approach specified in [4]. To complete the exercise students were required to submit their score along with screenshots of their LME experience.

One of the authors collated the scores from the 50 successfully completed SUS questionnaires (one was returned blank) and calculated the mean average score to be 70.05. A score above 70 is considered an above average score according to [2].

### SMAT: SHARC mobile authoring tool

**Fig. 7:**
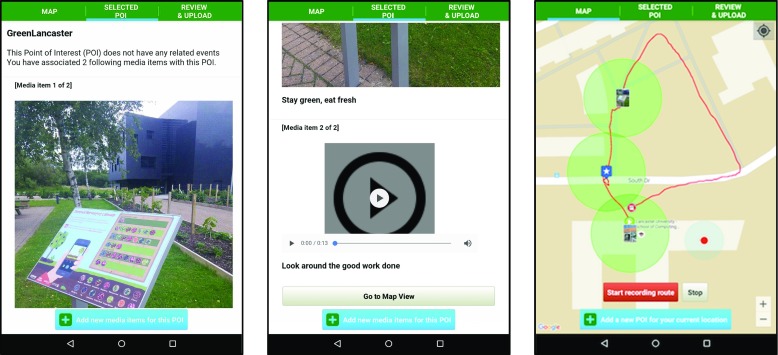
Illustrative screenshots of SMAT showing content produced during an expert evaluation that took place on Lancaster university campus in August 2015

The SMAT Android app has been developed in order to support the requirement for mobile authoring (identified earlier in section 2.2.1). An early version of the SMAT tool is presented in [9]. The UI of the current version of SMAT (presented here) has been significantly revised following an additional expert evaluation that took place in August 2015. Figure 7 shows three illustrative screenshots of SMAT.

In common with SMEP, the SMAT mobile app is designed to support offline use. It has been designed to provide a reduced set of features compared to SLAT with the intention that SLAT can be used to provide additional editing of an experience created by SMAT if required. The functionality supported by SMAT comprises: creating and publishing a LME, creating and recording a route (using the devices GPS), creating POIs and associating media items to created POIs by capturing media (e.g. audio, video) in-situ.

The SMAT application has undergone two expert evaluation sessions between July and August 2015. Both trials took place on Lancaster University campus and involved experts in mobile interaction design. During field usage of the app in the trials all wireless connectivity was disabled on the Nexus 7 tablets being used, and experts published their LMEs (via Wi-Fi connectivity) on returning to the main Computing department building. Feedback from the expert evaluations prompted a simplification of SMAT’s tab based UI and the redesign of UI buttons to make their function more apparent and their appearance more salient. In common with SMAT, SMEP also supports interaction logging.

### SPET: SHARC public exploration tool

**Fig. 8:**
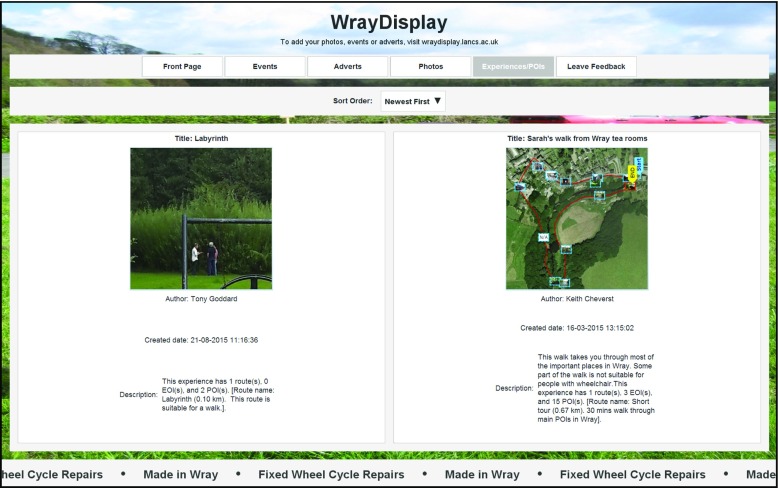
Screenshot illustrating how a user (resident or visitor) can interact with a *WrayDisplay* in order to browse published LMEs

**Fig. 9:**
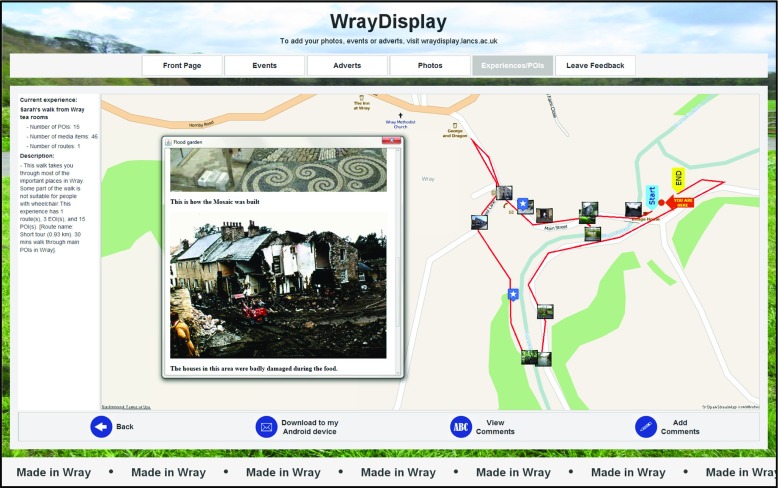
Screenshot illustrating how a user (resident or visitor) can interact with a *WrayDisplay* in order to view a LME and download it to their Android phone or tablet

The SPET component has been developed to support the browsing of published LMEs. Within the *WrayDisplay* software the SPET component enables users (residents and visitors) to browse LMEs associated with Wray (see Fig. 8) and to view routes and related media associated with a selected LME (see Fig. 9).

The summaries of available LMEs include a description (which can, for example, include accessibility information such as wheelchair access) and an illustrative image (see Fig. 8). The user can then select a specific LME in order to be shown a map view presenting details such as the route taken by the LME and the location of associated POIs (see Fig. 9).

### Summary

In this section we have presented the key components of the SHARC2.0 framework. The SLAT, SMAT and SMEP components have all received multiple expert evaluations and, in particular, SMEP was evaluated in the intended place of deployment in order to ensure strong reliability and usability for the field trial described in the following section.

## Field trial evaluation

At this stage of the research our aim for the field trial evaluation was to gain insights and understanding into the way in which visitors and residents would respond and react as they consumed the LME using the SMEP app while walking through the village.

Our decision to conduct a field trial was chosen in order to generate findings with greater ecological validity through more natural use of the system than would have been the case with a lab-based study [50] in which significant simulation would have been required. Indeed, in describing the role of field studies within HCI research [5] describe how: “*a set of users who are asked* (*often implicitly*) *to use the system* ‘*naturally*’ *outside the laboratory the system was designed in*”.

Furthermore, our approach to analyzing the qualitative data captured from the field study (i.e. transcripts of the audio recordings obtained from participants) has followed a grounded theory approach [19, 20] as opposed to using a model such as TAM (Technology Acceptance Model) [11] as a particular focus. In regards to analyzing the qualitative data from the field study, we carried out open coding of the transcripts in order to identify and develop a set of emergent themes (see section 4.2).

The field trial involved a LME called *Sarah*’*s Walk* that was based on a route and audio content contributed by a local historian (referred to in this article using the pseudonym ‘Sarah’) and which also included content supplied by residents. The trial took place in Wray in early May 2015 during a vintage car rally event.

The following sub-sections describe the preparation of the LME used in the field trial and the findings that arose from the field trial.

### Preparation of the ‘Sarah’s Walk’ LME

#### Circular walking route provided by local historian

The walking route that featured in the field trial was developed following a semi-structured interview and guided walk that took place with a local historian (with specialist expertise in 19th-20th Century history) in July 2013. During the interview with two researchers (both of whom are authors of this article), the historian was informed about the project’s aim and intention to produce a LME relating to the local history of Wray. Sarah gave a positive response to the concept, commenting: “*Yeah*, *have you seen the bus shelter with the flood sign on it*? … *Cause I thought in a place like this*, *if you have a series of points where you have a little bit of text and a photo and it says underneath*: ‘*if you*’*ve got a mobile then connect to see more photographs of what this used to look like here*’. *Then*, *people can stand on the street and look at them*…’*while over there the bridge swept away*”.

**Fig. 10:**
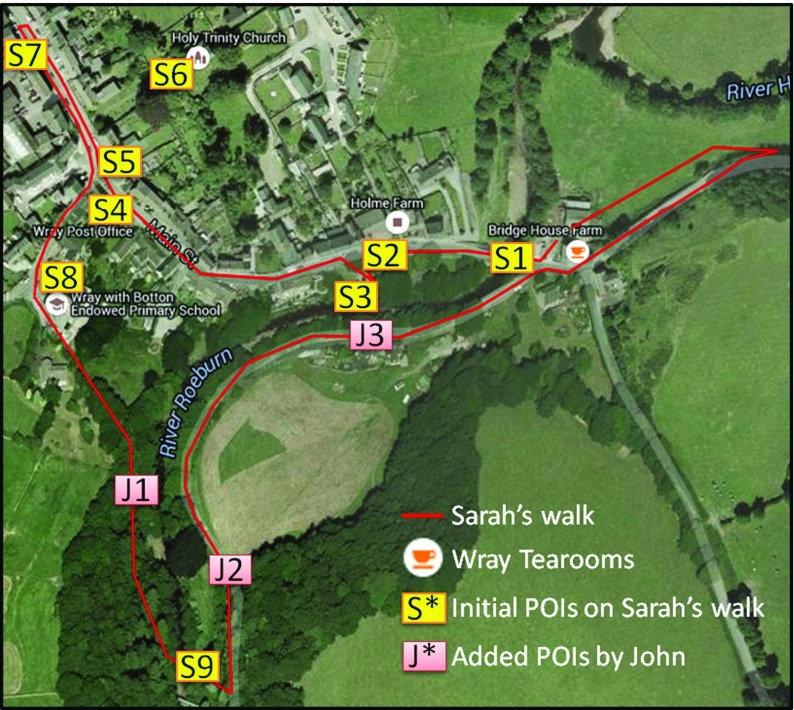
Map illustrating the route of *Sarah*’*s Walk* and associated POIs

Following the interview the historian proceeded to take the researchers on a 40 min guided walk of the village. The circular route encompassed nine POIs and covered 1.7 km (see Fig. 10).

During the walk, Sarah commented on the POIs as they were reached and explained their significance to Wray’s history. The commentary was recorded by dictaphone and the two researchers took photos of POIs and their features that were specifically addressed in the historian’s commentary. For example, the first POI encountered on the tour (see S1 in Fig. 10) was *Wray Bridge* POI involved a five second audio clip.

The *Sarah*’*s Walk* LME was created by the authors using SLAT and incorporated the nine POIs covered in the walk. Additional image content of historic photos from the *WrayDisplay* content archive was also added to supplement the audio recordings of Sarah and the photos taken during the walk. Three EOIs were created to reflect the themes focussed on during the walk, these were *Wray Flood*, *Millennium Celebration* and *Scarecrow Festival*.

#### Contribution of media to Sarah’s walk LME during design workshop

In order to gather the opinion of villagers regarding the developed LME a design workshop was held prior to the day vintage car rally. The workshop took place on 30th April 2015 and participants (responding to a post to Wray’s Facebook page) comprised five residents and two of the article’s authors. While a range of issues were discussed during the workshop, given the focus of this article we concentrate here on the contribution of media by one of the residents.

**Fig. 11:**
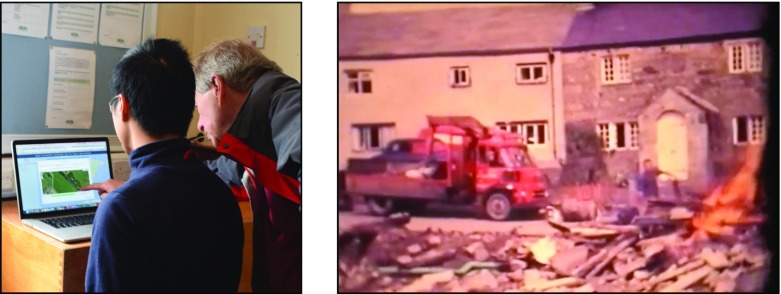
Resident using SLAT during the workshop (*left*) and still of video showing aftermath of Wray Flood that was contributed by resident during the workshop

During the workshop, participants were given the opportunity to view *Sarah*’*s Walk* LME using the SLAT tool (see Fig. 11, left) and also to try out the experience using SMEP (running on a Nexus 7 tablet). During the workshop one of the resident used SMEP and, encouraged by its use, suggested that we include some video material that she had discovered which showed some of the recovery operations following Wray flood. The resident showed the video (video still shown below in Fig. 11, right) which was shot using an 8 mm Cini Film camera.

**Fig. 12:**
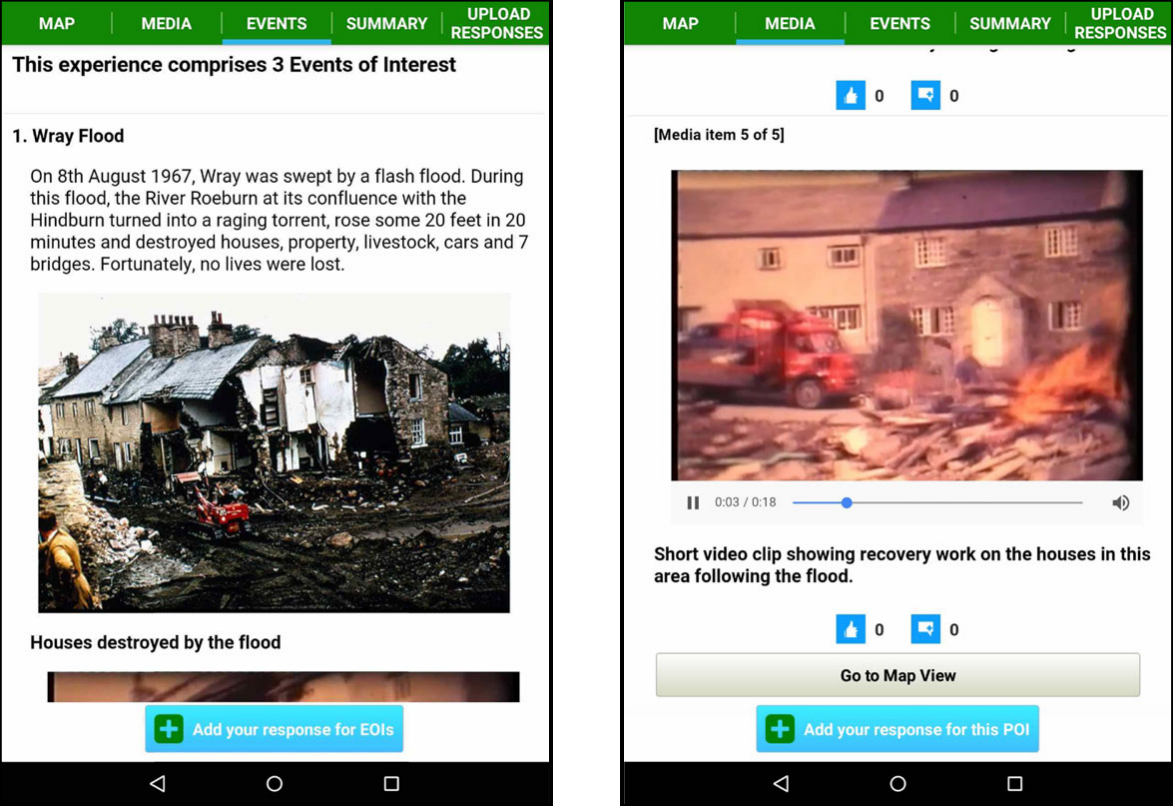
Illustrative screenshots of SMEP used during the Field Trial

The length of the video was over five mins in length and so it was agreed to produce a shorter ten second clip that would be included at the *Flood Garden* POI and as a media item associated with the *Wray Flood* EOI. Figure 12 shows illustrative screenshots relating to the *Wray Flood* EOI and *Flood Garden* POI.

Following the contribution of content made during the workshop *Sarah*’*s Walk* LME comprised 40 media items across nine POIs (see Table 1).

**Table 1:** Media associated with each POI used in *Sarah*’*s Walk* POI

POI	Media: Text	Media: Image	Media: Audio	Media: Video
Wray Bridge (S1)	3	3	0	0
Bus Shelter (S2)	1	2	1	0
Flood Garden (S3)	1	3	1	1
Post Office (S4)	1	2	0	0
Queen Victoria Jubilee Lamp post (S5)	1	2	1	0
Wray Church (S6)	1	3	0	0
Village Hall (S7)	1	2	0	0
Wray School (S8)	1	4	1	0
Kitten Bridge (S9)	1	2	1	0

### Use of SMEP during the field trial

On the day of the vintage car rally (3rd May 2015) the weather was inclement with a great deal of rain in the morning. Despite this, one trial session with a resident was completed in the morning and three sessions with visitors in the afternoon. Sessions lasted between 45 and 50 min. Table 2 summarises the participants involved in each session and media responses made. In all sessions, participants completed an informed consent form and were told that they would not be financially rewarded for taking part in the trial but could withdraw at any time.

**Table 2:** Participant and response details relating to use sessions in the Field Trial

Session	Description	Resident/Visitor(s)	Added media to the LME	Details media added
Session 1	Single Male (late 60s)	Resident	Yes	1 text, 3 photos, 2 audio
Session 2	Single Male (late 20s)	Visitor	Yes	1 test, 3 photos, 1 audio, 1 video
Session 3	Father (late 30s) and teenage Daughter	Visitors	Yes	1 photo
Session 4	Father, Mother (both mid 30s) and Two Young Sons	Visitors	No	N/A

Participants were provided with an Asus Google Nexus 7 tablet (16GB version) running the SMEP app. The Nexus 7 tablet was chosen because of its popular form factor and because it typically received updates to the Android OS in a timely manner. The SMEP app running on the tablet had already been used to download *Sarah*’*s Walk* LME which meant that all locative media content associated with the LME was stored on the device. Furthermore, the Google Maps tiles of Wray were cached on the device and the mobile player app signed into one of the test Dropbox accounts associated with the project. Participants were then given a short (approximately one minute) briefing about using SMEP, e.g. how to add responses. Sessions began and ended at the village hall. On returning to the village hall any responses made during the session (e.g. new POIs or media responses) were uploaded via Wi-Fi and accepted (by the authors) to become a new part of *Sarah*’*s Walk* LME.

Participants were encouraged to inform the authors (two of whom accompanied participants during each session) of any issues they encountered while using SMEP. In more detail, authors were able to answer queries regarding the interface and record and observe how SMEP was used. The authors also prompted participants for their understanding of the system if such comments were not forthcoming, e.g. understanding the use of push-bashed notifications when entering a trigger zone. In order to avoid potential bias the authors were careful not to make comments or statements that would encourage the participants into making unduly positive comments regarding the system (however, the potential positive bias caused by the authors being present cannot be discounted and is discussed in section 6.1).

Following the field trial the one of the authors transcribed the audio recordings captured during field-trial sessions and two of the authors (both with previous coding experience) carried out collaborative open coding of the transcripts (with a third author validating) in order to produce a set of emerging themes following a grounded theory approach to analysing the qualitative data [19, 20]. The following six themes emerged: i) *Response to feature of the environment*/*landscape*, ii) *Response to locative media*, iii) *Usability*, iv) *Co*-*authoring and Organic growth of Content*, v) *Suggestions for future features*, and, vi) *Technical enquires*. Examples of comments relating to these themes are presented in the following sub-sections that describe each of the four field trial sessions.

#### Use and contribution to LME by long term resident of wray

Despite the rain in the morning, one of the residents helping to marshal the Vintage car rally was willing be a participant. The participant, John, was a retired man in his late-sixties and a long time resident of Wray.

The session lasted 50 min and John explained that he used Facebook to keep up-to-date with village events and that his family had lived in Wray for generations.

John appeared very engaged with the experience of following *Sarah*’*s walk*. During the session he created three new POIs and contributed five media items (see Table 2). For example, approximately half way through the experience, we approached a natural play area for children and John responded to this place by stating: “*ah*, *here is the forest school. Shall we take a picture of that*?”.

**Fig. 13:**
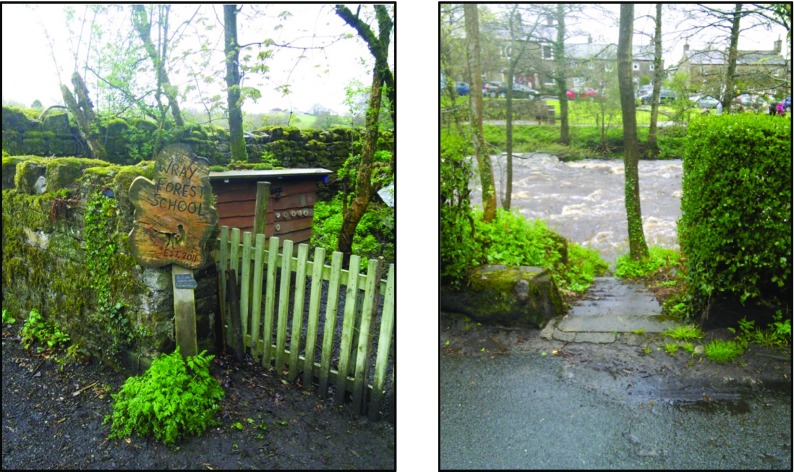
Resident used SMEP to create two new POIs with associated images: *Forest School* (left) and *Stepping Stones* (right)

John used SMEP to ‘Respond to current location’ and called the new POI “*Forest School*”. He then added a picture to it (see Fig. 13, left). This action and comment relates to the *Response to feature of the environment*/*landscape* theme and a further example occurred a further 200 m along the route where we encountered a section of river where John commented: “*This is a walking route across the river which may have predated the bridge but I don*’*t know that for sure*…”

John then created a new POI (calling it *Stepping Stones*) and also contributed a 15 s audio description detailing the value of the garden for groups of children brought to Wray on school trips from poor inner city neighbourhoods.

When asked about the push-based approach and the audio notifications for indicating new content John commented: “*When you get used to it* [*the audio alerts*] *you*’*ve stopped looking at the thing*… *so you can just carry on walking* … *and so you need those alerts*.” (a response relating to the *Usability* theme).

Next we approached *Wray Bridge* and after viewing the locative media content he described his understanding of the role that the Bridge had played in the flood and the resulting devastation:

“This bridge is the cause of quite a lot of the damage. Because it actually wasn’t destroyed … the arch held… the tree jammed under the bridge so the water lifted… so what it did was … it ripped through the houses and blew the houses out. So this bridge was key to what happened at the time of the flood.”

He then recorded a slightly shorter version (30 s) as an audio response to the *Wray Bridge* POI. The next POI encountered was the *Flood Garden* POI and on playing the video (Fig. 12, right), John exclaimed (while pointing at a man driving a tractor in the video clip): “*Wow that*’*s brilliant* – *who found that*? … *Now I bet that if you showed that to Bill Bowman he would know who that bloke was*” (a comment relating to both the *Response to locative media* theme and the *Co*-*authoring and Organic growth of Content* theme).

#### Use by single male visitor in late twenties

Paul, a single male in his late 20s, was highly computer literate and when presented with the tablet actually asked if he could install SMEP on his own Android tablet (this being one of several questions he asked during the session that related to the *Technical enquires* theme). We agreed to this and he successfully managed to download and install SMEP and then download the LME on his own tablet. Before downloading the LME he commented that he would prefer to know the size of an LME before downloading it onto his own device (this is a feature that was subsequently implemented).

When prompted to comment on the push-based approach and triggering of media, Paul commented:

“I thought the trigger point was quite good actually because it happened just as you got there…”

On occasion Paul also reacted to images that he was shown (relating to the *Response to locative media* theme), especially regarding the flood:

“… picture of the flooding - wow that’s spectacular isn’t it”.

Paul also made six responses to *Sarah*’*s walk* including a photo with a caption response to the *Stepping stones* POI added by John earlier (see Fig. 13, right and Fig. 15).

When asked about the nature of adding responses to the experiences he responded with a the following positive comment (related to the *Co*-*authoring and Organic growth of Content* theme): “*Well its going to be a very organic thing isn*’*t it really* … *I can see it being a really good experience for the users*…” but later expressed a more concerned comment relating to the same theme: “*but is it going to be overwhelming if lots and lots of people are contributing*”.

#### Use by father and teenage daughter

**Fig. 14:**
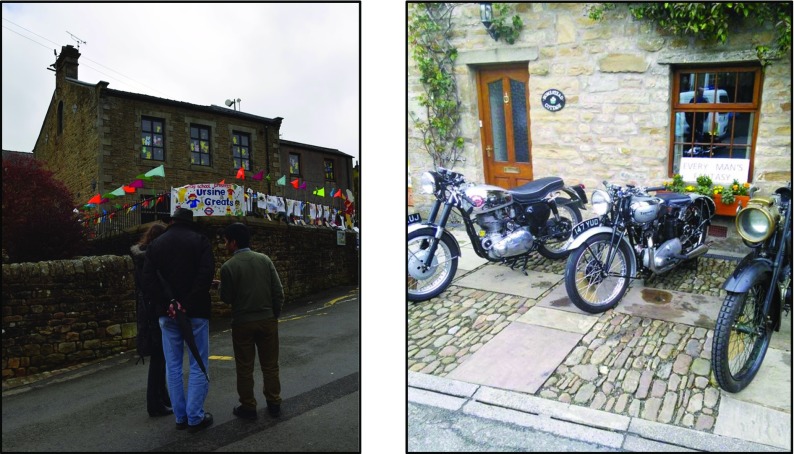
Father and daughter (accompanied by one of the authors) approaching *Wray School* POI (*left*) and the photo of vintage motorbikes contributed by the daughter as a new POI (*right*)

The session involving a father and his teenage daughter (see Fig. 14, left) lasted 48 min. Despite having come to Wray the previous year, he appeared enthusiastic about returning to the village and commented: “*I like going round these quaint little places and seeing how it was*…”

The teenage daughter held the tablet during most of the session but would regularly show her father the content displayed. As POIs were approached and associated media pushed they would each speak out the names of the POIs and scroll through the media items. At one point along the route the daughter noticed some vintage motorcycles and decided to create a new POI with an associated photo (see Fig. 14, right).

Towards the end of the session the father suggested that the system could incorporate a game (a comment relating to the *Suggestions for future features* theme): “*something the kids will do to keep them occupied*, *my youngest daughter might get a bit fed up so it will keep her occupied* … *like quizzes*, *you*’*ve got to find certain scarecrows to get a letter and make the word up as you go around*.”

#### Use by mother and father and two young sons

During this final session the father took control of the tablet while the mother attended to the young boys. Consequently, the father (who ran a digital marketing company) provided most of the feedback.

He made two comments of relevance to this article. The first concerned his desire for a certain structure in the content presented for each POI. For example, on viewing the content associated with *Wray school* (which did not actually contain details on when the school was actually built) he commented: “*a picture and then some quick stats would have been useful* …*Built in this year and currently used for this*” (a comment relating to both the *Response to locative media* and *Response to feature of the environment*/*landscape* themes.)

On reaching the *Flood Garden* POI and noticing the availability of the archive video footage the father commented: “*Its great that I can play something*, *that definitely should be there*” (again this comment related to both the locative media and the feature, i.e. the Flood Garden).

Towards the end of the session, the father made a comment (relating to the *Suggestions for future features* theme):

“I was just thinking yeah, it would be good if there was something that was integrated into an app for facebook and any interaction you made on here, whether it’s a comments or anything, it’s kind of, when you got back on to wifi it’s just a sync and it would auto sync, so like if I could upload a video, from say I’ve got a camera on this, and I want to upload a video of this, and I could just click upload, so when I get back home, throw this down on the sofa and it just syncs automatically”

At the end of the session the father made a pertinent comment (relating to the theme of *Usability*) based on his professional experience in digital marketing:

“I think the key is just dumbing it down - seriously making it as simple as possible - I think a lot of that is based on the pop-ups but as well actually adding the information as an input, so if you wanted to add a response, then you would give them options, so are you adding to a POI, adding a video … then just give them a step-by-step process”

### Summary of field trial

The field trial involved an LME that featured a circular walking tour provided by a local historian and was supplemented by content from the *WrayDisplay* content archive and additional video material. The latter was contributed by a resident during a design workshop in the village prior to the field trial. The field trial itself provided a further opportunity for use and media contribution by one of Wray’s residents. In this case, the long term resident used SMEP to create new POIs to supplement *Sarah*’*s Walk* LME and also contributed image and audio content.

Our aim for the trial was to gain insights and understanding into the way in which visitors and residents would respond and react as they consumed the LME in-situ. A grounded theory approach was followed to analyse the qualitative data which involved the use of collaborative open coding leading to six emergent themes.

In the following section, we return to the SHARC2.0 framework to illustrate the propagation of media through personal cloud storage.

## Propagation of media

**Fig. 15:**
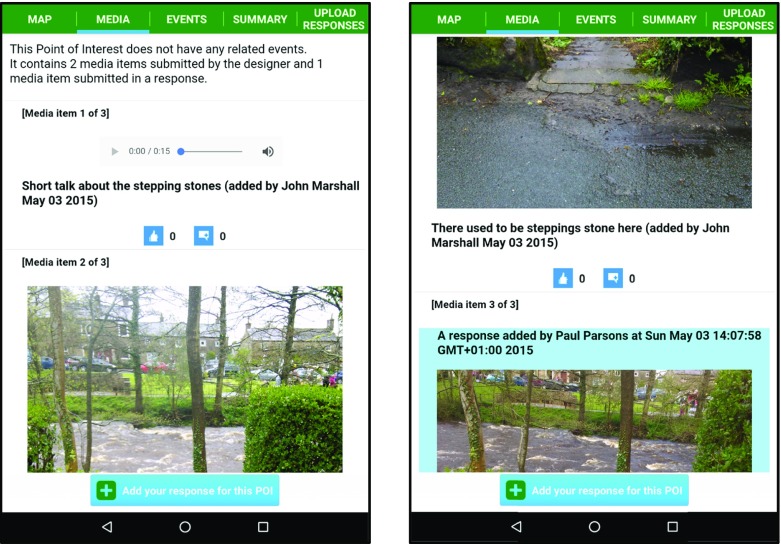
Illustrative screenshots of SMEP showing the media tab view (including Paul’s media response) that would appear to on approaching the *Stepping Stones* POI

In this section, we return to the *Stepping Stones* POI and associated responses that arose during the field trial in order to further illustrate the design and workings of the SHARC framework components. Recall that this POI was created by resident John while walking the route of *Sarah*’*s Walk* and that a subsequent response was added by a visitor, Paul. The response was fully added to *Sarah*’*s Walk* once the authors accepted the response via SLAT. Subsequent visitors, on approaching the *Stepping Stones* POI, would be presented with the new content as shown in Fig. 15.

**Fig. 16:**
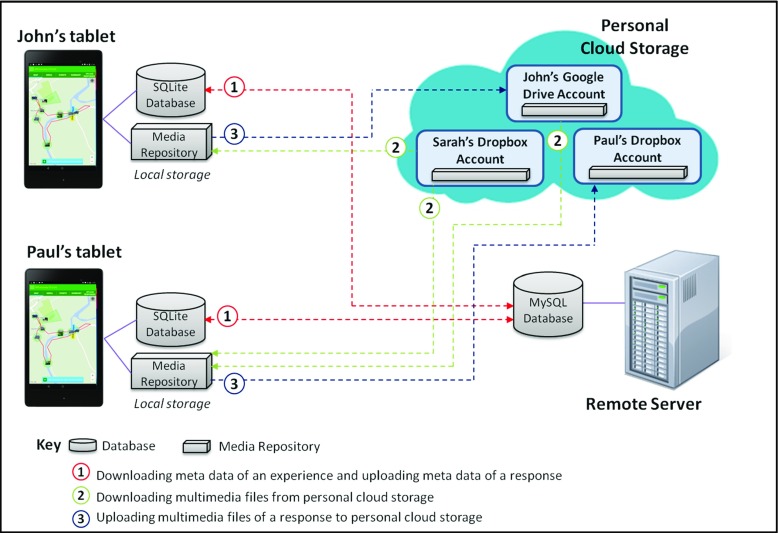
Exchange of data between components during use of the SMEP app

The overall architecture of the system and the key interactions between the end-user devices and underlying services is shown in Fig. 16. A MySQL database is used to store meta data relating to every item of media and the experience(s) to which it is associated (see the red path numbered 1 in Fig. 16). Meta data associated with an experience is cached in a local SQLite database at the end user’s device to enable off-line operation during the experience (which, if updated, will later be synchronised with the main MySQL database). Before an experience begins all associated media is downloaded from cloud storage to a media repository on the user’s device to enable off-line operation (see the green path numbered 2 in Fig. 16). New media associated with an experience by a user is temporarily cached in the media repository until appropriate network connectivity allows it to be uploaded to the user’s own personal cloud storage account (see the blue path numbered 3 in Fig. 16).

The following steps illustrate the propagation across the personal cloud storage owned by Sarah, John and Paul. Note that in the field trial the moderation step was not actually performed by Sarah (who could not attend) but by the authors.

Step 1) At the village hall, John has *Sarah*’*s Walk* LME downloaded onto his tablet.
  Multimedia files associated with the LME are downloaded from Sarah’s Dropbox personal cloud storage and saved onto his tablet.
  Metadata associated with the LME is downloaded from the MySQL database on the remote server and saved into an SQLite database on his tablet.
Step 2) On arriving at the location of the *Stepping Stones*, John adds a response to create a new POI which included a short audio narrative (15 s, 26 KB, mp3 format) and a photo (2.35 MB, jpg format).
  The audio clip and the photo are stored locally on his tablet
  Metadata for the response is stored locally in the SQLite database on his tablet.
Step 3) After completing the LME, John returns to the village hall, and uploads his response via the Wi-Fi in the village hall.
  The audio clip and photo are uploaded from his tablet to his Dropbox personal cloud storage.
  Metadata associated with the response are uploaded from the SQLite database on his tablet to the MySQL database on the remote server.
Step 4) Sarah (the designer of *Sarah*’*s Walk* LME) receives a notification email about John’s response. She logs into SLAT and accepts John’s response to create a new POI and the associated media.
Step 5) Paul arrives at the village hall and downloads the LME.
  Multimedia media files are downloaded from Sarah’s Dropbox personal cloud storage and saved onto Paul’s tablet
  Multimedia media files (the audio clip and photo in John’s response) are downloaded from John’s Dropbox personal cloud storage and saved onto Paul’s tablet.
  Metadata associated with the experience are downloaded from the MySQL database on the remote server and saved into an SQLite database on his tablet.
Step 6) At the *Stepping Stones* POI, Paul listens to John’s audio narrative while viewing John’s photo. Paul then adds a response to this POI with a photo.
  The photo is stored locally on his tablet.
  Metadata for the response is stored locally in the SQLite database on his tablet.
Step 7) Once the experience is finished, Paul returns to the village hall, logs into SMEP with his Dropbox credentials and uploads his response.
  The photo is uploaded from his tablet to his Dropbox personal cloud storage.
  Metadata for the response is uploaded from the SQLite database on his tablet to the MySQL database on the remote server.

Again, Sarah receives a notification email about Paul’s response and accepts it. Consequently, the multimedia files associated with the LME are stored across Sarah, John, and Peter’s personal cloud storage.

**Fig. 17:**
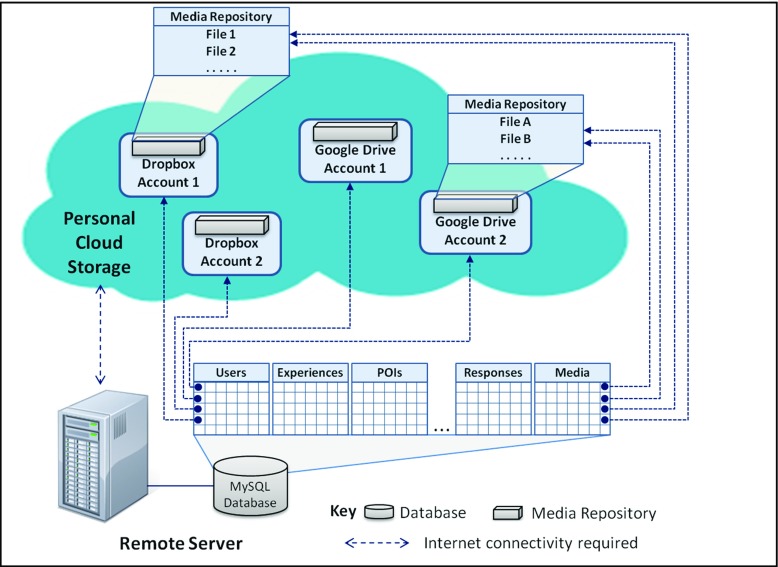
Utilisation of Dropbox and Google Drive cloud storage within the SHARC2.0 framework

In the field trial Dropbox only was utilised however the SHARC2.0 framework currently supports both Dropbox and Google Drive. Figure 17 illustrates the key aspects of the information architecture within the SHARC2.0 Framework and its utilisation of Dropbox or Google Drive cloud storage. In more detail, a set of MySQL tables hosted on a central server are used to store the details of personal cloud storage accounts associated with users of the system, and to store details of specific pieces of media hosted within these accounts. The applications utilising the SHARC2.0 framework communicate directly with the central server to access the MySQL tables, and use appropriate APIs to communicate directly with the cloud storage services to upload and retrieve media. In order to access Dropbox and Google Drive accounts from a third party applications (such as those within the SHARC framework) the OAuth 2 protocol [27] is used, whereby unique access tokens are generated and used in place of username/password authentication (these access tokens are stored within the central database).

Both DropBox and Google Drive provide access to their services for third party developers through web services. These web services provide similar mechanisms for listing, downloading and uploading files but differ slightly in the way that calls are made to the web services and, for example, the ways that file types are identified and file locations are expressed. DropBox and Google Drive provide their own programming language specific APIs for a range of development platforms which simplifies access to the web services. The SHARC applications make use of the Android APIs for both cloud services.

## Discussion

The approaches adopted for the design of the SHARC2.0 framework and the field trial evaluation in Wray raise a number of points for discussion and these are presented in the following sub-sections.

### Evaluation approach and potential bias in the field study

It is important to note the limitations of the field trial carried out and the potential of researcher bias between participants and the authors present during the field trial. While every effort was made not to encourage participants to provide overly positive feedback regarding the system, the fact that the researchers were present means that such an effect could have occurred. Furthermore, as a think aloud method was being followed, the authors prompted participants for verbal explanation if they were observed having difficulty using the system but such an approach can increase the anxiety of participants and potentially effect their behaviour with the system. The authors present also provided verbal assistance if participants struggled with a particular feature and prompted participants to comment on the push based media approach if feedback on this aspect was not forthcoming. When analysing the qualitative data from the field trial two authors carried out collaborative open coding in order to avoid lone researcher bias while a third author carried out validation.

The nature of the shared walking activity involved in the field trial, i.e. between researcher and participant, also appeared to have an effect. In more detail, the walking together aspect of the field trial appeared to produce a phenomena similar to that discussed in ‘Fieldwork on Foot’ [35] where Lee and Ingold describe how “*Sharing or creating a walking rhythm with other people can lead to a very particular closeness and bond between the people involved*”. During the field trial, the act of walking with John, through his home village, certainly appeared to facilitate a more open and collaborative context and also related to Lee and Ingold observation that “… *such conversations taking place as we walk show how temporality in walking can be shifting and unsettled*: *thinking and perceiving the past*, *present and future*, *and combining them in references to routes*” [35].

### Open approach to authoring and sustainability

The open approach to authoring LMEs in-situ is one of the novel aspects of our approach and one of the emergent themes that arose from the field trial was that of *Co*-*authoring and Organic growth of Content*. It is certainly the case that further studies need to be run in order to understand more fully the issues and complexities involved in supporting co-authoring, yet the field trial did reveal initial understanding and positive and negative aspects. For example, during the field-trial session with Paul, he positively commented on the “organic” nature of the co-authoring feature and how this could result in “really good experience for the users” while also acknowledging the potential for it to become “overwhelming”.

Another comment relating to this theme arose during the field trial while walking with Wray resident John (and prompted by him noticing a smell of wild Garlic):

“… the advantage I guess is, that you’d get someone like Sarah for the history and someone [else] for nature who could go and say ’well we’re here now in Spring because I can smell wild garlic, if you’re here in Autumn you won’t smell that, you’ll see this and you’ll see that’ … this is the future isn’t it”

One issue associated with the open authoring approach relates to perceived effort to moderate responses associated with a given LME. The approach adopted in the trial was to provide a large degree of openness, i.e. one in which all users could attempt to add new POIs, associated media items, etc. and it would be the responsibility of the LME’s owner to moderate these. In Wray, the local historian was not prepared to take on this potential burden not because of a lack of interest but because of other time commitments including authoring a book. However, another resident who has been involved in a community project to lay high-speed broadband cable into the village is planning to create a LME that illustrates to residents and visitors the extent of the cable channels and a collection of ‘before and ‘after’ photos. She has stated that she would “*prefer folk to be able to add to it*” (e-mail correspondence, February 2016) and is prepared to moderate such possible additions.

Less ‘open’ approaches to co-authoring are also possible. For example, the ability to add new POIs to a LME could be restricted to members of a given group, e.g. a local history group. In this situation, the owner of the LME could effectively be the history group (sharing a common e-mail address and their own cloud storage accounts). In this case, the decision of the group could be to disable the requirement for moderation given trust in what its own members may chose to contribute.

### Personal cloud storage and sustainability

The general issue of sustainability relates to and cuts across the previous points of discussion. In general, the importance of considering the media storage aspect of sustainability when designing for community-based deployments in the wild is discussed in [37] and [52]. Our current approach effectively reflects the aims of [45] in attempting to support sustainability through local ownership, and in particular, through an approach that utilises personal cloud storage. Adopting such an approach was motivated by the difficulty of effectively managing the handover of content with the community when a particular ‘research in the wild’ project comes to an end [52].

The handover of content could be particularly problematical for the SHARC project where a given LME can have content contributed by multiple users. However, by utilising personal cloud storage each contributor maintains ownership and effective control over their contributed media. As a result of this approach, when the SHARC research project draws to an end, the researchers will discuss with residents the possibility of them running their own remote server in order to sustain access to the LMEs that have been developed for Wray.

A natural consequence of using personal cloud storage is the potential of media included in LMEs becoming unavailable. This may happen for a range of reasons such as content being accidentally (or purposely) deleted, renamed or moved, access configurations being changed, cloud storage accounts being closed, and so forth. Several possibilities exist for reacting to and guarding against unavailable media such as backups, periodic checking, media expiry dates, notifications, alternative media, and purges. However, this is a complex area we are keen to explore with involvement from our users in order to reach a solution that balances the need maintain a positive relationship between contributors and the system, and the need to ensure the integrity of LMEs is maintained over the long term.

## Conclusions and future work

In this article we have presented the design and evaluation of the SHARC2.0 framework for supporting the authoring and consumption of Locative Media Experiences (LMEs).

The framework has a number of key features, namely:

i. Supporting the mobile authoring and consumption of LMEs in-situ and in environments with poor cellular data coverage, as typified by rural locations.
ii. Enabling LMEs that can organically grow through multiple authored responses, including responses to an encountered feature or place resulting in a new POI, responses to an existing POI or responses to an individual media item.
iii. Utilising personal cloud storage (currently Dropbox and Google Drive are supported) to facilitate local ownership of content and distributed ownership for those LMEs that comprise multiple authored responses.
iv. Integration of LMEs within an existing community-based situated public display system, i.e. using *WrayDisplays* to support the advertisement and browsing of LMEs and enabling LMEs to be downloaded via the displays.

The Field trial evaluation presented took place in Wray and involved both residents and visitors consuming and responding to a LME relating to Wray’s local history. This LME contained multimedia content contributed by members of the community including historic photos (taken from the *WrayDisplay* content archive), audio-clips (from a local historian and village residents) and archive video (contributed by a resident during a design workshop). It was the latter video that resulted in the most compelling engagement during the field trial. The video clip related strongly to a key event in the village’s history and clearly associated with the street location in the village in which it was triggered.

During the field trial, responses were made by those consuming *Sarah*’*s Walk* resulting in an LME that was effectively co-authored by both residents and visitors. The support for, and demonstration of, such in-situ co-authoring represents a novel contribution in the field of locative media. Furthermore, the rich findings obtained from the field trial provide insights into both the potential value of supporting the co-authoring of locative media (in terms of enabling visitors and residents to engage with history and historical content in new ways) but also the challenges associated with such an approach, e.g. the burden on users that have moderation responsibilities and the implications that this has for long term participation and sustainability.

Finally, our approach has explored the use of cloud technologies, and in particular the utilisation of personal cloud storage, in enabling the distributed ownership of media associated with co-authored LMEs. Utilising personal cloud storage in this work was motivated by a desire to give contributors a sense of ownership relating to their specific contributions within the context of the wider system and content. This approach was also chosen as contributors are investing both their own time initially and their own resources over the longer term in the system, as free cloud storage plans (on Dropbox and Google Drive for example) have finite amount storage capacity and contributors may even have paid storage plans. We see these issues of ownership and investment as being important to maintaining engagement, participation, and sustainability over the longer term.

In terms of future work, the authors are holding a second design workshop with residents in April 2016 in order to facilitate the creation of additional LMEs relating to the village (e.g. an LME associated with a recent community project laying high-speed broadband cable into the village) and to discuss further the potential issues, obstacles and opportunities. The outcomes from this workshop will then inform the design of a further field trial during Wray’s 2016 May Day festival.
